# Association Between Increased Body Mass Index (BMI) and Atopic Dermatitis in Children Attending a Tertiary Referral Center: A Case-Control Study

**DOI:** 10.7759/cureus.60770

**Published:** 2024-05-21

**Authors:** Adelina-Maria Sendrea, Sinziana Cristea, Carmen Maria Salavastru

**Affiliations:** 1 Pediatric Dermatology, Carol Davila University of Medicine and Pharmacy, Bucharest, ROU; 2 Dermatology Reseach Unit, Colentina Clinical Hospital, Bucharest, ROU; 3 Pediatric Dermatology, Colentina Clinical Hospital, Bucharest, ROU; 4 Integrated Drug Development Consulting, Certara USA, Inc., Princeton, USA

**Keywords:** scorad, bmi, overweight, obesity, atopic dermatitis

## Abstract

Background

Atopic dermatitis (AD) and obesity represent chronic diseases, with growing worldwide prevalence, that rely on a common pathophysiological background: perpetual inflammation. Moreover, AD is considered more and more to be a beyond-the-skin disease with various associated comorbidities. This study aimed to investigate a potential link between overweight/obese status and AD in children.

Methods

A case-control study was performed on 130 AD patients and 130 exact age and sex match controls that attended the Pediatric Dermatology Department of Colentina Clinical Hospital. Based on the weight (in kilograms) and height (in centimeters), the body mass index (BMI), and the corresponding age and gender percentiles were assessed in both groups; study participants were divided as normal-weight, underweight, overweight, or obese. AD severity was evaluated using the Scoring Atopic Dermatitis Index (SCORAD), and quality of life impairment was assessed with the Dermatology Life Quality Index (DLQI). Descriptive statistics, t-tests, and logistic regression with odds ratios (OR) and associated 95% confidence intervals (CI) were used for data analysis.

Results

A statistically significantly higher BMI was identified in the AD group compared to controls (p=0.027). The relative risk for overweight/obese status in the AD group compared to controls was three times more frequent (OR 3.61, 95% CI 1.45-10.3, p<0.01). Additionally, the increased BMI in the AD group correlated significantly with disease severity as determined by SCORAD (p<0.05), with a relative risk for overweight/obese status in the moderate-severe AD subgroup being 20 times more frequent as compared to mild AD (OR 20.4, 95% CI 6.53-90.7, p<0.001).

Conclusions

To our knowledge, this is the first study to evaluate the correlation between AD and BMI in Romanian children. Statistically significant correlations between increased BMI, AD development, and AD severity in children were identified in our study population. This study's small sample size and single-center study design represent possible limitations. Additional, larger, multicentric studies are required to establish a more precise correlation between AD and obesity. Physicians should be aware of this potential association in order to perform obesity screening in AD children for more appropriate multidisciplinary management of such patients.

## Introduction

Atopic dermatitis (AD) represents a relapsing, inflammatory condition characterized by pruritic erythematous papules and plaques with morphologic features and distribution depending on the patient's age, associated with skin xerosis. It represents the most common dermatological condition among pediatric patients, with variable prevalence among different geographical locations (from 0.65% in Tunisia to 34% in Sweden) [1]. Despite being predominantly a pediatric condition, it can also affect adults. AD pathogenesis relies on an interplay between inflammatory, genetic, immunological, and environmental factors, being a Th2-driven inflammatory condition [2]. Although it is widely considered a dermatological condition, in recent years more and more evidence has brought to light the fact that this disease might be associated with various comorbidities, among which cardiovascular disease, metabolic syndrome, and obesity might be encountered, along with mental health disorders (i.e., depression, anxiety, attention deficit hyperactivity disorder, or autism spectrum disorder), bone disorders (i.e., osteoporosis and fractures), or skin infections [3].

The potential association between AD and obesity could be attributed to various factors, starting with the common pathophysiological background of perpetual inflammation, which is driven and maintained by various elements such as adipokines (e.g., adiponectin), fatty acid-binding proteins, proinflammatory cytokines, or hormones (e.g., leptin) [4,5]. Moreover, obesity contributes to epidermal barrier dysfunction and has some shared risk factors with AD, such as poor diet and certain alterations in the intestinal microbiome [6]. Currently, data regarding the co-occurrence of AD and obesity or overweight status are controversial and debatable; some studies support this association [7,8], while others don’t [9]. However, studies have found a geographically variable association between obesity and AD in both children [10] and adults [11]. Moreover, obesity seems to be associated with an increased severity of AD in the pediatric population [6]. The goal of this study was to assess the potential relationship between increased body mass index (BMI) and AD in a pediatric population.

## Materials and methods

We performed a retrospective, case-control study on 130 patients with AD aged one-17 years old and 130 exact age and sex-match controls who were referred to the Pediatric Dermatology Department of Colentina Clinical Hospital, Bucharest, Romania, East Europe, from January 2022 until January 2024.

The inclusion criteria for the case group (i.e., the AD group) were represented by children aged more than one year old with clinically active AD without any known personal medical history or systemic treatment. For the control group, the same number of children with the exact age and sex match were selected, excluding children with any known personal medical history (including atopic background).

AD was diagnosed based on the clinical examination using the UK’s Working Party diagnostic criteria [7], and disease severity was assessed using the Scoring Atopic Dermatitis Index (SCORAD), with cases being divided into mild (SCORAD <25), moderate (SCORAD 25-50) and severe (SCORAD >50) [8]. Quality of life impairment was evaluated in the AD group using the Dermatology Life Quality Index (DLQI) and its children’s version for children younger than 16 years [9]. Anthropometric parameters were evaluated in all participants: weight was measured in kilograms and height in centimeters; body mass index (BMI) was calculated as shown in equation 1:

$BMI (\frac{kg}{m^{2}})=\frac{weight(kg)}{height^2 (m^2 )}.$

The corresponding BMI and percentiles (pctl) for age and sex were determined using the CDC BMI Percentile Calculator for Child and Teen [10] and the Ped(z) Pediatric Calculator with CDC/WHO data [11] for children older than two years and younger than two years, respectively. The resulting BMI for age and sex percentiles were used to determine the patient’s nutritional status as underweight (pctl <5), normal weight (5≤ pctl <85), overweight (85≤ pctl <95), and obese (pctl ≥95).

Descriptive statistics data were summarized as mean and standard deviation (SD), including the median and entire data range for AD cases and the control group. For AD cases, the data was further stratified based on disease severity. A comparison between the patient characteristics of AD cases and controls was not required, as groups were matched for age and sex. A paired t-test on the exact age and sex match was used to estimate the association between BMI and AD. Odds ratios and associated 95% confidence intervals (95% CI) were calculated by means of a logistic regression to assess whether patients in the AD group have a higher relative risk of being overweight or obese as compared to the control group, and whether patients with moderate or severe AD have a higher relative risk of being overweight or obese as compared to patients with mild AD. The threshold for statistical significance was set at p <0.05. The data exploration and analysis were performed in the R version with RStudio version 2023.03.0 Build 386.

## Results

Study population characteristics

As AD cases and control groups were exact age and sex match, the mean (SD) of age in both groups was 5 (± 3.62) years, with a range between one and 17 years. Early childhood age group (2-<6 years) was most commonly encountered (43.1%), followed by middle childhood (6-<12 years) (28.5%), toddlers (1-<2 years) (23.1%), and adolescents (12-17 years) (5.4%). The same distribution between males and females was kept in both AD and control cases, with a slight predominance of female patients (56.9%) as compared to males (43.1%) (Table 1).

**Table 1:** Study population characteristics. SD: standard deviation; AD: atopic dermatitis; CV: coefficient of variation; Min: minimum; Max: maximum; kg: kilograms; m: meters

	AD (N=130)	Controls (N=130)
			Age (years)		
Mean (SD) [CV%]	5.00 (3.62) [72.4%]	5.00 (3.62) [72.4%]
Median [Min, Max]	3.88 [1.00, 17.6]	3.88 [1.00, 17.6]
Weight (kg)		
Mean (SD) [CV%]	22.6 (14.6) [64.7%]	20.7 (10.9) [52.6%]
Median [Min, Max]	18.0 [8.50, 105]	18.5 [8.00, 66.0]
Sex		
Female	74 (56.9%)	74 (56.9%)
Male	56 (43.1%)	56 (43.1%)
BMI (kg/m^2)		
Mean (SD) [CV%]	16.6 (3.25) [19.5%]	15.8 (1.95) [12.3%]
Median [Min, Max]	16.0 [11.1, 34.3]	15.6 [12.0, 22.9]
Nutritional Status		
Underweight	7 (5.4%)	9 (6.9%)
Healthy weight	94 (72.3%)	113 (86.9%)
Overweight	18 (13.8%)	6 (4.6%)
Obesity	11 (8.5%)	2 (1.5%)
Age Group		
Toddler (1-<2 years)	30 (23.1%)	30 (23.1%)
Early childhood (2-<6 years)	56 (43.1%)	56 (43.1%)
Middle childhood (6-<12 years)	37 (28.5%)	37 (28.5%)
Adolescents (12-17 years)	7 (5.4%)	7 (5.4%)

The control group consisted of 130 children without any history of atopic background, diagnosed with various skin infections and infestations: tinea capitis (29.2%), warts (23.8%), molluscum contagiosum (23.1%), skin candidiasis (14.6%), scabies (3.8%), impetigo (3.1%), and herpes simplex virus infection (2.4%).

The mean BMI was comparable between AD cases and control groups, as the majority of patients are categorized as having a normal weight. However, the variability in the BMI of the AD case groups is higher, with an SD of 3.25 as compared to 1.95 in the control group, indicative of a larger BMI range in this group. This is mostly driven by the increased prevalence of overweight and obese patients in the AD case group as compared to the control group (Table 1).

BMI-AD correlation

**Figure 1:**
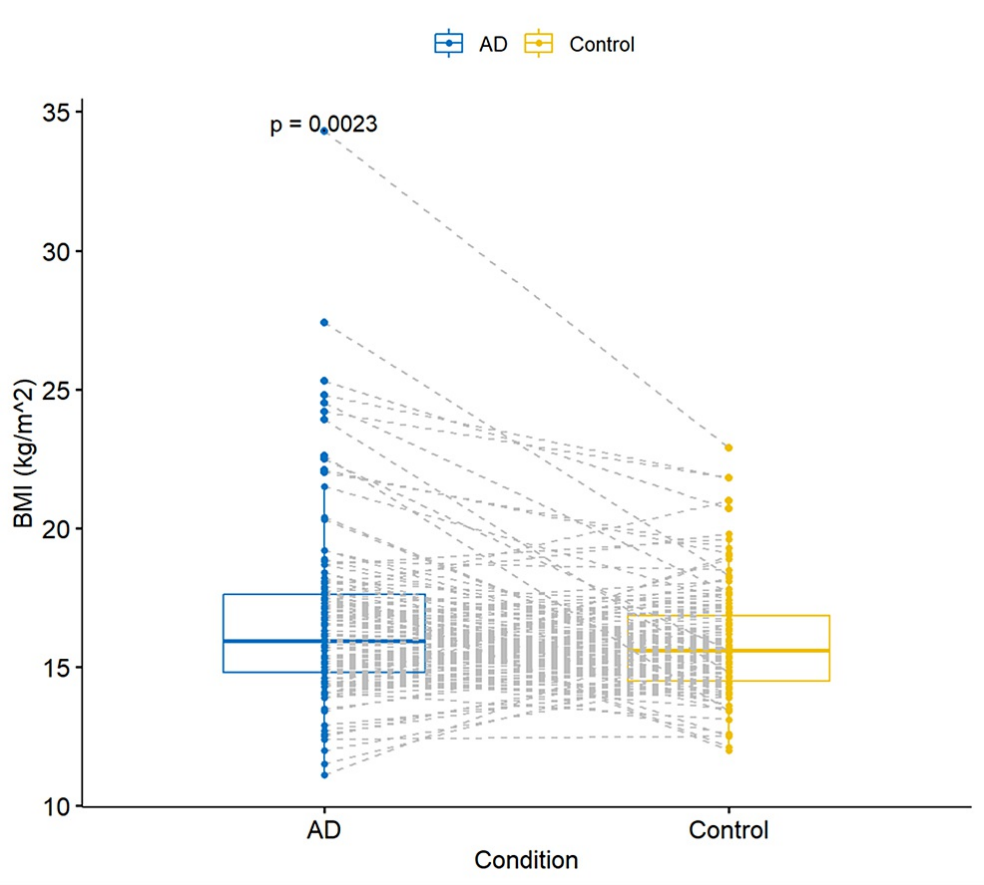
Boxplots comparing the BMI of the AD group and controls by performing a paired t-test. Boxplots show the mean and interquartile range (IQR) of the data. Individual dots show the individual measurements of BMI for each group. AD cases are represented in blue and controls in yellow. The gray dotted lines represent the direct pairing between the AD and control patients. BMI: body mass index; p: p-value

A paired t-test was performed between control patients and their matching age and sex counterparts in the AD group. This showed a statistically significantly higher BMI in the AD case group as compared to the control group (p=0.0023) (Figure 1).

This correlation is also supported by an increased weight (22.6 ± 14.6 vs. 20.7 ± 10.9) and frequency of overweight and obese patients (22.3% vs. 6.1%) in the AD cases group as compared to the control group (Table 1).

Moreover, the relative risk of being overweight in the AD group compared to controls was more than three times more frequent (OR 3.61, CI 1.45-10.3, p <0.01). Similarly, the relative risk of being obese in the AD group compared to controls was more than six times more frequent (OR 6.61, 95% CI 1.72, 43.4, p<0.05). The 95% CI for OR is broad, indicating a low level of precision; however, neither includes the null value of OR=1.

BMI-SCORAD and CDLQI correlation

In the study group, the AD severity distribution identified predominantly mild cases (55.4%), followed by moderate (38.5%) and severe (6.1%). The mean SCORAD for all patients with AD was 25 (± 16.7), while for each severity subgroup it was as follows: mild cases 13.9 (± 5.57), moderate cases 33.7 (± 9.18), and severe cases 69.7 (± 12). The mean age increased with AD severity as follows: mild AD, 4.46 (± 3.33) years old; moderate AD, 5.64 (± 4.03) years old; and severe AD, 5.91 (± 2.91) years old. A slightly higher representation of female patients was noted in the mild (56.9% vs. 43.1%) and moderate (58% vs. 42%) severity groups, while in the severe one the sex distribution was equal. Nonetheless, the sex distribution across AD severity groups remains balanced (Table 2).

**Table 2:** Atopic dermatitis group characteristics. SD: standard deviation; AD: atopic dermatitis; CV: coefficient of variation; Min: minimum; Max-maximum; kg: kilograms; m: meters; SCORAD: Scoring Atopic Dermatitis; DLQI: Dermatology Life Quality Index

	Mild (N=72)	Moderate (N=50)	Severe (N=8)
				Age (years)			
Mean (SD) [CV%]	4.46 (3.33) [74.6%]	5.64 (4.03) [71.5%]	5.91 (2.91) [49.2%]
Median [Min, Max]	3.25 [1.00, 16.2]	5.17 [1.08, 17.6]	5.79 [1.83, 9.75]
Weight (kg)			
Mean (SD) [CV%]	20.1 (10.4) [51.9%]	26.3 (19.3) [73.3%]	21.4 (7.49) [35.1%]
Median [Min, Max]	17.5 [8.50, 67.3]	21.0 [9.20, 105]	21.5 [12.0, 30.0]
Sex			
Female	41 (56.9%)	29 (58.0%)	4 (50.0%)
Male	31 (43.1%)	21 (42.0%)	4 (50.0%)
BMI (kg/m^2)			
Mean (SD) [CV%]	15.7 (1.81) [11.6%]	18.1 (4.31) [23.9%]	16.6 (2.48) [14.9%]
Median [Min, Max]	15.4 [11.5, 22.1]	17.3 [11.1, 34.3]	17.8 [12.0, 18.7]
Nutritional status			
Underweight	3 (4.2%)	3 (6.0%)	1 (12.5%)
Healthy weight	66 (91.7%)	24 (48.0%)	4 (50.0%)
Overweight	3 (4.2%)	13 (26.0%)	2 (25.0%)
Obesity	0 (0%)	10 (20.0%)	1 (12.5%)
Age group			
Toddler (1-<2 years)	22 (30.6%)	7 (14.0%)	1 (12.5%)
Early childhood (2-<6 years)	29 (40.3%)	24 (48.0%)	3 (37.5%)
Middle childhood (6-<12 years)	18 (25.0%)	15 (30.0%)	4 (50.0%)
Adolescents (12-17 years)	3 (4.2%)	4 (8.0%)	0 (0%)
SCORAD			
Mean (SD) [CV%]	13.9 (5.57) [40.1%]	33.7 (9.18) [27.2%]	69.7 (12.0) [17.2%]
Median [Min, Max]	13.0 [1.20, 28.6]	33.7 [1.20, 49.2]	70.7 [49.0, 90.7]
DLQI			
Mean (SD) [CV%]	5.63 (4.45) [79.2%]	11.1 (6.18) [55.9%]	17.4 (6.16) [35.5%]
Median [Min, Max]	4.00 [1.00, 25.0]	9.50 [1.00, 26.0]	18.5 [9.00, 25.0]

**Figure 2:**
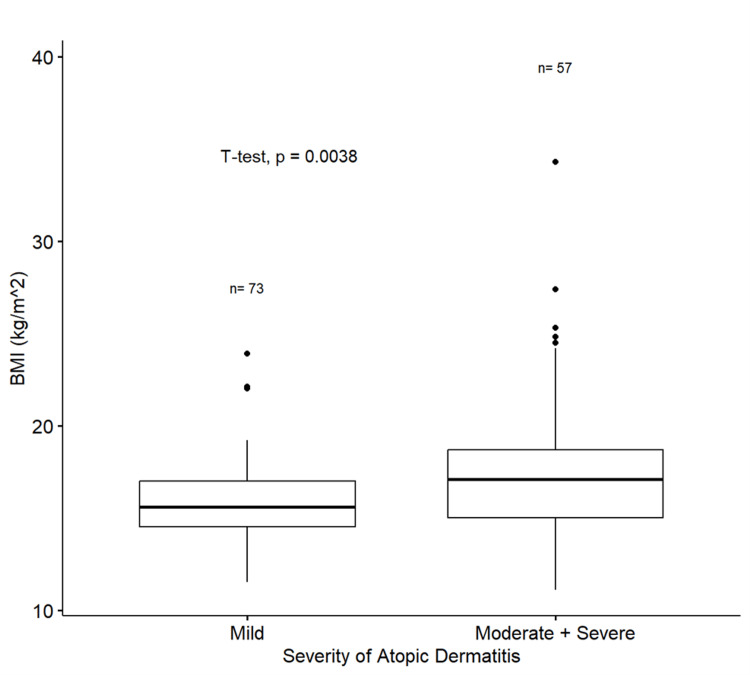
Boxplots comparing the BMI of the mild AD subgroup and moderate-severe AD subgroup by performing a t-test. Boxplots show the mean and interquartile range (IQR) of the data. The whiskers show the entire range of data and the dots represent the outliers. BMI: body mass index; p: p-value

A significant increase (t-test, p <0.05) was identified in the mean BMI of the moderate-severe AD subgroup compared to the mild one (Figure 2).

This is further supported by the higher prevalence of overweight (51%) and obese (32.5%) patients in the moderate-severe subgroup compared to the mild AD subgroup (4.2% overweight, none obese) (Table 2). Moreover, the relative risk of being overweight or obese was about 20 times more frequent (OR 20.4, 95% CI 6.53-90.7, p<0.001) in the moderate-severe AD subgroup as compared to the mild AD subgroup. The 95% CI for OR is broad, indicating a low level of precision; however, neither includes the null value of OR=1.

The mean (SD) DLQI value for all AD patients was 8.44 (+/- 6.29), which was proportional to disease severity (Table 2). No significant correlation was identified between increased BMI and quality of life impairment in the AD group (p=0.9, following linear regression).

## Discussion

In this study, we compared pediatric patients with AD with age- and sex-matched controls and identified a statistically significant higher frequency of an increased BMI (corresponding to overweight and obese status) in the AD group compared to controls. Moreover, the increased BMI correlated statistically significantly with the AD severity (as evaluated by SCORAD).

The correlation between being overweight or obese and having AD has been studied frequently in the past decades, yielding conflicting findings. Currently, there is an ongoing debate concerning the association between these two diseases and the nature of their relationship (i.e., does obesity cause AD, is it vice versa, or are we facing a bidirectional connection?). A meta-analysis performed on 30 studies identified a correlation between overweight/obesity and the higher occurrence and severity of AD, with geographical differences (in North America and Asia, not Europe) [12]. Some case-control studies revealed potential links between increased BMI and AD, analyzing various pediatric populations. A study in the United States (involving pediatric patients aged one to 21 years old) identified that long-term obesity with early onset is linked to an increased prevalence and severity of AD [13]. Another study from Spain (children up to 14 years old) identified a greater prevalence of overweight and obesity in AD patients compared to controls, with specifically higher BMI values in two age groups (zero to two years old and 12-14 years old) and a correlation with AD severity in nine to 12 and 12-14-year-old groups [14]. In the current study, we did not observe a higher prevalence of overweight or obesity in a specific age group with AD, but across all groups, as compared to the control group. An Egypt-based study (children aged five-18 years) showed significantly elevated BMI values in AD patients compared to controls and additional connections with fasting glucose, serum insulin, triglycerides, total cholesterol, and C-reactive protein levels [15]. Another study from India (pediatric patients aged two to 18 years old) revealed a significant rise in the prevalence of metabolic syndrome in AD children, with a strong correlation between AD severity and elevated BMI [16]. However, a study analyzing Chilean children of all age groups found no disparity in obesity rates between AD patients and controls [17].

Various possible links might interconnect overweight/obesity with AD. However, the exact mechanism that is connecting these two entities has not been identified yet. Both represent chronic conditions that rely on a perpetual inflammatory background. Various proinflammatory cytokines secreted in the adipose tissue (e.g., tumor necrosis factor (TNF)-alpha, interleukin (IL)-6) [12,18] have a pathogenic role in AD [19]. Moreover, some adipokines with central roles in promoting obesity are found in connection with AD. Leptin, mainly secreted by the white adipocytes, exhibits proinflammatory properties, including T helper 1 response promotion, cytokines upregulation (e.g., TNF-alpha, IL-6, interferon-gamma, IL-2) or proliferation and activation of T cells (CD4+ and CD8+) and monocytes [13,20,21]. Elevated serum levels of leptin have been found in children with immunoglobulin E (IgE)-mediated AD, but also in teenagers and adults with AD, irrespective of their BMI [22]. Adiponectin, however, found in the skeletal muscles, with a decreased expression in obesity, has anti-inflammatory activity, arising from inhibitory effects on macrophages, keratinocytes, and T helper 17 cells [21]. Furthermore, it enhances the production of structural proteins involved in epidermal barrier maintenance, like filaggrin, loricrin, and involucrin [21], known to be defective in AD. Decreased serum levels of adiponectin have been identified in AD patients compared to controls [23], and serum levels correlated negatively with AD severity in adult patients [24]. Some AD-related symptoms might lead to obesity development: chronic pruritus determines sleep disorders, and active eczema lesions represent a reason for avoiding physical activity, both of which contribute to weight gain [25]. Additionally, obesity increases transepidermal water loss and decreases stratum corneum lipids [12], both of which are key factors in AD development.

## Conclusions

To our knowledge, this is the first study to assess the association between AD and BMI in a Romanian pediatric population. In our study population, we identified that children with AD are more likely to be overweight or obese, and there is a positive correlation with AD severity. 

Potential strengths of the study are represented by the clinical criteria-based AD diagnosis performed by dermatologists with experience in pediatric dermatology and the anthropometric measurements performed by physicians and not self-reported by the patients. Possible limitations of this study are represented by the small sample size and study design (single center).

Further larger, multi-centric studies are needed to establish a more accurate potential connection between AD and obesity that might serve as potential prognostic factors and a better treatment choice in selected patients.
